# Social Phenotypes of Autism Spectrum Disorders and Williams Syndrome: Similarities and Differences

**DOI:** 10.3389/fpsyg.2012.00247

**Published:** 2012-07-30

**Authors:** Kosuke Asada, Shoji Itakura

**Affiliations:** ^1^Center for Baby Science, Graduate School of Psychology, Doshisha UniversityKyoto, Japan; ^2^Japan Society for the Promotion of ScienceTokyo, Japan; ^3^Department of Psychology, Graduate School of Letters, Kyoto UniversityKyoto, Japan

**Keywords:** autism spectrum disorders, Williams syndrome, Autism Diagnostic Observation Schedule, face processing, social cognition, sociability, communication

## Abstract

Autism spectrum disorders (ASD) and Williams syndrome (WS) both are neurodevelopmental disorders, each with a unique social phenotypic pattern. This review article aims to define the similarities and differences between the social phenotypes of ASD and WS. We review studies that have examined individuals with WS using diagnostic assessments such as the Autism Diagnostic Observation Schedule (ADOS), cross-syndrome direct comparison studies, and studies that have individually examined either disorder. We conclude that (1) individuals with these disorders show quite contrasting phenotypes for face processing (i.e., preference to faces and eyes) and sociability (i.e., interest in and motivation to interact with others), and (2) although the ADOS and a direct comparison study on pragmatic language ability suggest more deficits in ASD, individuals with WS are similarly impaired on social cognition and communicative skills. In light of these results, we discuss how cross-syndrome comparisons between ASD and WS can contribute to developmental theory, cognitive neuroscience, and the development and choice of clinical treatments.

## Introduction

Autism spectrum disorders (ASD) and Williams syndrome (WS) both are neurodevelopmental disorders. ASD are a group of pervasive developmental disorders usually first seen in childhood, characterized by impairment of social interaction and communication, and by restricted, repetitive, and stereotyped behaviors (American Psychiatric Association, 2000). Approximately half of individuals with ASD have a mild to profound intellectual disability and the other half have cognitive abilities within the normal range of intelligence, while a minority have intelligent quotients well above normal (Joseph, 2011). WS is a rare genetic neurodevelopmental disorder, which is caused by a microdeletion of chromosome 7q11.23 (Ewart et al., 1993). This deletion can be confirmed by fluorescence *in situ* hybridization (FISH) genetic test. Most individuals with WS have a borderline to moderate intellectual disability (Mervis and John, 2010).

Autism spectrum disorders and WS have been described as “opposite” disorders in terms of their social behavior (e.g., Jones et al., 2000). However, there are very few detailed comparisons of the social phenotypes of ASD and WS, including aspects such as social cognition and communicative skills (see Tager-Flusberg et al., 2006 for a review of face recognition and emotion processing with ASD and WS). A better understanding of the similarities and differences between these two disorders could provide insights into gene-brain-behavior relationships. Recently, some genetic studies have begun to identify genes related to both ASD and WS (Feyder et al., 2010; Sanders et al., 2011). Therefore, from the perspective of behavioral genetics, it is important to determine detailed social phenotypes of ASD and WS. In this article, we will review the social phenotypes of these disorders with respect to the following domains: diagnostic assessments, face processing, social cognition, sociability, and communicative skills. After that, we will discuss how a cross-syndrome comparison between ASD and WS can contribute to developmental theory, cognitive neuroscience, and the development and choice of clinical treatments.

## Diagnostic Assessments

Earlier studies have reported concurrence of autism and WS (Reiss et al., 1985; Gillberg et al., 1991; Gillberg and Rasmussen, 1994). For example, Gillberg and Rasmussen (1994) provided case reports of four children who had concurrent autism and WS within 60 WS cases registered in their clinic. However, there have been no systematic studies using standardized assessments, and therefore how many individuals with WS would have ASD and how many deficits related to ASD they would have is unknown.

Recently, studies of individuals with ASD and those with WS have been done using the Autism Diagnostic Observation Schedule (ADOS; Lord et al., 1999). The ADOS is a structured interaction designed to assess play, reciprocal social interaction, and communication skills, and to diagnose ASD across a range of ages. Lincoln et al. (2007) assessed both children with WS and age- and IQ-equivalent children with autistic disorder using the ADOS Module 1. Among their sample, the DSM-IV (American Psychiatric Association, 1994) criteria placed 20% of the children with WS in the ASD range. Of the 20% group, half met criteria for autistic disorder, and half for pervasive developmental disorder not otherwise specified (PDD-NOS). The ADOS algorithm placed 10% of the children with WS in the ASD range. Of the 10% group, half met criteria for autism, and half for autism spectrum. They found that many children with WS showed some problems in using pointing (55%), initiating joint attention (50%), and showing an object to another person (65%), but few showed symptoms involving shared enjoyment in interaction (0%), facial expressions directed to others (5%), and quality of social overtures (10%). In addition, they reported that the WS group showed fewer problem behaviors in all of the ADOS items than the autistic disorder group, and a discriminant analysis of ADOS behaviors classified 100% of the cases consistent with their original diagnosis.

On the other hand, Klein-Tasman et al. (2009) emphasized overlaps of ASD with WS, compared with Lincoln et al. (2007). They examined children with WS, autism, PDD-NOS, and mixed etiology non-spectrum developmental disabilities, using the ADOS Module 1. The ADOS algorithm classified the children with WS as autism (10%), autism spectrum (40%), and non-spectrum (50%). Moreover, the WS group showed fewer sociocommunicative abnormalities than the autism group, but about the same as the PDD-NOS group. Klein-Tasman et al. (2009) explained that their results differed from the Lincoln et al. (2007) study because the level of difficulties in their WS sample was more severe than that of Lincoln et al. Also, they did stricter assessments as their sample was well matched with the targets of the ADOS Module 1 compared to those of Lincoln et al. The higher rate at which individuals with WS were categorized as ASD in the Lincoln et al. (2007) and Klein-Tasman et al. (2009) studies might be related to the expanded notion of autism as a spectrum disorder. Klein-Tasman et al. (2007) reported the detailed data of these children with WS.

These studies taken together, using the ADOS, found a certain percentage of individuals with WS showed problem behaviors indicative of ASD, and they were indeed classified as ASD, although the extent of sociocommunicative deficits was less with WS. As Lincoln et al. (2007) pointed out, the quality of some social behaviors (e.g., quality of social overtures) indeed is different between ASD and WS. However, taking into consideration that half of the children with WS were categorized as ASD (Klein-Tasman et al., 2009), we should keep in mind some autistic traits exist within WS. Exploring which social phenotypes are similarly impaired could contribute to understanding the mechanisms of these two disorders. In the following sections, we will take a closer look at similarities and differences between ASD and WS in each social phenotype.

## Face Processing

Most of the direct comparison studies between ASD and WS are with respect to face processing. Riby and Hancock (2008, 2009a,b) examined the way individuals with ASD or WS viewed social stimuli by using eye-tracking techniques. Riby and Hancock (2008) showed photographs of human actors to individuals with autism or WS and typically developing individuals matched for chronological age or non-verbal ability. The individuals with autism spent less time than the control groups viewing actors’ faces, but the opposite pattern was found for individuals with WS. A detailed analysis showed that, while individuals with autism spent less time viewing the eyes, individuals with WS spent more time on the eye region than the control groups. Similar results were reported using static cartoon images and movies containing human actors or cartoon characters by Riby and Hancock (2009b). The distinct difference between ASD and WS regarding interest in faces also was found in other types of eye-tracking studies. Riby and Hancock (2009a) examined how individuals with autism or WS viewed scrambled pictures containing faces and pictures of scenes with embedded faces. They reported that individuals with autism showed fewer and shorter fixations on faces, while individuals with WS showed prolonged fixations on faces.

The way that individuals with ASD or WS view faces relates to their face matching skills. Riby et al. (2009) provided individuals with autism or WS unfamiliar face matching tasks, which required the participants to use eyes and mouth cues. Individuals with autism in general performed relatively poorly on these matching tasks, and showed particular deficits when they needed to use the eyes region. On the other hand, individuals with WS showed the typical pattern of performance, with greater accuracy using the eyes than the mouth region.

In addition, Riby et al. (2008) directly compared the face processing skills of ASD and WS individuals. They showed that children and adolescents with WS performed better on processing expressions of emotion (pointing to an image of the person depicting an expression verbalized by the researcher, and matching different faces showing the same expressions) and the direction of eye-gazes (pointing to the face that was looking at the participants, and matching the persons looking in a named direction) than their counterparts with autism matched for non-verbal ability and chronological age. However, Lacroix et al. (2009) reported a different finding with emotion recognition. They used an emotion identification task which required participants to point out the person who expressed a certain emotion among three pictures, and found that children with WS performed worse than children with autism and typically developing children (these groups were matched for verbal mental age).

Taken together, individuals with WS show preference to faces and eyes to a greater extent even than typically developing individuals, while individuals with ASD do not. In addition, although more studies are needed, individuals with WS perform better on some face processing tasks (i.e., matching faces and processing eye-gaze directions) than those with ASD, but mixed findings were obtained regarding emotion recognition.

## Social Cognition

Social cognitive ability is thought to be one of the most impaired domains of ASD, and considerable effort has been made to research false belief understanding. In Baron-Cohen et al.’s (1985) seminal study, performance on a false belief task was worse by children with autism than by children with Down syndrome or typically developing children. Another study showed that the acquisition of a first-order false belief with autism was considerably delayed. The verbal mental age at which 50% of participants passed the false belief task was 4 years for typically developing children, and 9 years 2 months for those with autism (Happé, (1995). A recent study using the eye-tracking version of the false belief task suggests this difficulty is found even among adults with Asperger syndrome (Senju et al., 2009).

Although there are many studies revealing the impairment of false belief understanding by those with ASD (e.g., Perner et al., 1989; Leekam and Perner, 1991; Leslie and Thaiss, 1992), relatively few studies of this sort have been done regarding WS. Tager-Flusberg and Sullivan (2000) used the false belief task and compared the performance of children with WS to that of age-, IQ-, and language ability-matched children with Prader–Willi syndrome (a genetic disorder resulting from the loss of paternal gene expression at chromosome 15q11-q13), and children with non-specific mental retardation. They showed that the number of WS participants who passed the task was significantly fewer than in the Prader–Willi syndrome group or the non-specific mental retardation group, suggesting impairment of false belief understanding with WS.

The finding of Tager-Flusberg and Sullivan (2000) is partly supported by a subsequent study using a different paradigm. Porter et al. (2008) used a non-verbal picture sequencing task that assesses understanding of false beliefs. They showed that individuals with WS performed worse than mental age-matched typically developing children. However, they also reported that this result was found only for a subgroup of WS, who had better verbal skills compared to their overall mental age.

The impairment of false belief understanding in WS might be surprising given that language promotes false belief understanding (e.g., Farrar and Maag, 2002) and vocabulary skills are not so impaired in WS (e.g., Bellugi et al., 2000; Brock, 2007). However, although data from individuals with ASD support the notion that vocabulary ability correlates with performance on a false belief task (Happé, 1995), we may assume, at least in some developmental disorders, that language is not enough for the acquisition of false belief understanding. This idea is supported by a recent study that revealed individuals with Asperger syndrome could not pass a non-verbal version of a false belief task, even if they have adequate verbal skills and indeed passed a traditional verbal false belief task (Senju et al., 2009). Furthermore, as discussed in the following sectionon joint attention development, this idea also is consistent with the notion that in ASD and WS, developmental pathways for language and social cognition are unique and relatively unrelated compared with typical development.

Joint attention skills are another aspect of social cognition that has been examined in ASD and WS. Joint attention is thought to be a precursor to complex social cognitive ability (Baron-Cohen, 1995), and there is a relationship between early joint attention behavior and performance on the false belief task (Charman et al., 2000). Deficits in joint attention behaviors with ASD have been found by a number of studies (Curcio, 1978; Mundy et al., 1986; Sigman et al., 1986; Baron-Cohen, 1989).

Mundy et al. (1986) used the Early Social Communication Scales (ESCS; Seibert and Hogan, 1982), a semi-structured observation session to assess a variety of communicative behaviors between the tester and children, with children with autism and children with mental retardation matched for chronological age and mental age. They found that, compared to children with mental retardation, children with autism showed less turn-taking behavior, and less eye contact with the tester. Also, Sigman et al. (1986) observed caregiver-child play interactions and found that, compared to chronological age- and mental age-matched children with mental retardation, children with autism less frequently displayed attention-sharing behaviors such as pointing to an object or showing/giving an object to the caregiver. Curcio (1978) found deficits in joint attention ability with autism, and revealed that non-verbal children with autism showed imperative gestures (i.e., using an adult to obtain an object, event, or activity), but not declarative ones (i.e., using an object to obtain the attention of an adult). Finally, Baron-Cohen (1989) examined comprehension and production of pointing, a key behavior of joint attention, in children with autism. He found that, unlike in control children, those with autism had difficulty both in comprehending and producing declarative pointing.

For individuals with WS, Laing et al. (2002) also used the ESCS (Seibert and Hogan, 1982). They revealed that toddlers with WS more frequently engaged in dyadic, face-to-face interactions, but less did so in triadic joint attention interaction (i.e., an interaction between the child, caregiver, and an external object outside of the face-to-face interaction) than mental age-matched typically developing infants. In addition to using the ESCS, in another experiment they examined comprehension and production of declarative pointing, and found that toddlers with WS had difficulty with both. The discrepancy between imperative and declarative communicative functioning, which was found with individuals with ASD, has not been clearly found with individuals with WS. However, recently, Asada et al. (2010b) found that children with WS clarified what they wanted when they were given the wrong object but not when their requests for objects were just verbally misunderstood, while typically developing children corrected others’ misunderstanding in both situations. Therefore, they suggested that the characteristic of ASD, that is, impaired declarative communicative functioning but relatively unimpaired imperative functioning, might be found regarding verbal communications of those with WS.

In addition, it appears there is a similarity in the developmental relationship of joint attention behavior and language production between individuals with ASD and WS. Carpenter et al.’s (2002) cross-sectional study has pointed out that, contrary to both typically developing children and children with developmental delays, there is a possibility that children with autism show a reverse developmental pattern, that is, they may produce referential language before engaging in joint attention behaviors. These findings are surprising, considering that with typical development, words are acquired partly through joint attention behaviors (e.g., Baldwin, (1995; Baron-Cohen et al., 1997). Itoh’s (2000) longitudinal study also demonstrated that, although this reverse pattern has been found with other developmental disorders, the gap between the onset of pointing behavior and language was not as long as with individuals with autism (mean gap between onsets: autism, 7.8 months; moderate or severe mental retardation, 3 months). This reverse developmental pattern also was found for WS, and the duration of the gap was almost the same as that for autism. Specifically, Mervis and Bertrand’s (1997) longitudinal study found that children with WS followed this reverse pattern, with a gap between onsets of 6 months. A delay in the onset of pointing, which is an early milestone of social cognitive development, compared to that of language, might suggest that verbal communication involving social cognitive ability (e.g., pragmatics) will suffer both with ASD and WS. Due to differences in research methodology, it is difficult to directly compare these findings. However, at least the developmental pathway of social communication in ASD and WS is similar, and it might be related to later social deficits in both disorders.

In sum, social cognitive ability (i.e., false belief understanding and joint attention) is severely impaired with ASD and also deficient with WS, although there is no direct comparison study between the two disorders regarding the level of difficulties. In addition, the relationship between the development of social cognition and language is unique for both ASD and WS, and it might lead to an atypical social communicative profile.

## Sociability

Studies have examined sociability in ASD and WS. Here, we review the degree to which individuals are interested in or motivated to interact with other people. The biggest difference between ASD and WS occurs in this area. Jones et al. (2000) gave the Salk Institute Sociability Questionnaire to parents of individuals with autism, WS, and Down syndrome, and to parents of typically developing individuals (each group was matched for chronological age). According to a qualitative analysis of the questionnaires, a parent of an individual with autism reported he needed a prompt to say hello and avoided people whenever possible. In contrast, a parent of an individual with WS reported that he was quite happy to meet people and asked a lot of questions. In addition, a qualitative analysis of the questionnaires revealed that individuals with WS were rated as most sociable, individuals with autism were rated as least sociable, and those with Down syndrome and typically developing individuals were rated between WS and autism.

Other studies have reported disinterest and insensitivity to social engagement in ASD. In a retrospective home video analysis, Baranek (1999) reported that children with autism needed more adult prompts to respond after their names were called and more of them showed social touch aversions, compared to children with developmental disabilities and typically developing children. In other home video analysis studies, Osterling and colleagues (Osterling and Dawson, 1994; Osterling et al., 2002) reported that children with ASD looked at others and oriented to their names less frequently than children with mental retardation or typically developing children. In addition, a study using observation of everyday school activities reported that children with autism, on average, initiated communication only three to four times per hour, and spontaneous communication was a relatively rare event for these children (Stone and Caro-Martinez, 1990).

In contrast to ASD, sociable traits of individuals with WS have been reported in various settings. Mervis et al. (2003) observed a scene in a hospital, and found that especially younger children with WS looked intensely at a medical staff, although none of the children in other groups (e.g., children with developmental delay) exhibited this behavior. Similarly, excessive looking at the experimenter by children with WS was reported during play and cognitive assessment (Jones et al., 2000). In addition, Jones et al. (2000) reported that, compared with chronological age-matched typically developing children, children with WS less frequently exhibited negative expressions, and if they occurred, the intensity of them was milder during a task in which the children and their parents were intentionally separated to observe their facial expressions. Recently, Dodd et al. (2010) observed approach behaviors toward strangers in play sessions, and found that pre-school children with WS were more willing to approach others than chronological age- or mental age-matched typically developing children. Only pre-school children with WS initiated interactions with strangers before the strangers noticed them, while none of the typically developing children did so.

Other studies also have found individuals with WS strongly motivated to interact with others. Jones et al. (2000) showed photographs of unfamiliar adult faces to individuals with WS and chronological age- or mental age-matched typically developing individuals, and asked them how much they would like to go up to each person and begin a conversation. Those with WS rated the faces more approachable than both comparison groups. This result was replicated by a study using the same stimuli as Jones et al.’s (2000) study (Martens et al., 2009). Frigerio et al. (2006) found that individuals with WS rated positive faces more approachable and negative faces less approachable than chronological age- or mental age-matched typically developing individuals. Adolphs et al. (2001) used the same approachability task as Jones et al. (2000), and found that high-functioning individuals with autism rated negative faces more approachable than typically developing individuals. Although we should be cautious whether this finding can be generalized to others with ASD, considering that individuals with ASD or WS tend to rate faces more approachable, this finding might indicate social cognitive deficits rather than a motivation to approach others.

Taken together, while individuals with WS have sociable traits and actively want to interact with others, individuals with ASD are relatively insensitive to others’ behaviors and are not so interested in engaging socially.

## Communicative Skills

In this section, we review communicative skills, mainly pragmatic language ability. Pragmatic language ability is broadly defined as the ability to use language in a social context for the purpose of communication. With ASD, pragmatic language is thought to be the most impaired among language abilities, while vocabulary and grammar abilities are relatively less impaired (Tager-Flusberg, 1993, 2000; Fein et al., 1996; Kelly, 2011; Tager-Flusberg et al., 2011). For example, vocabulary ability is a relative strength compared with other language abilities (Fein et al., 1996; Kjelgaard and Tager-Flusberg, 2001; Mottron, 2004). The developmental pathway for grammatical ability in autism follows that of typically developing children, although it is delayed (Tager-Flusberg et al., 1990). Indeed, the level of language abilities is variable across individuals with ASD, but among them pragmatic language ability is universally impaired (Kelly, 2011; Tager-Flusberg et al., 2011).

Some studies on pragmatic language ability have focused on narrative skills of individuals with ASD. Losh and Capps (2003) examined narrative skills of high-functioning children with autism or Asperger syndrome, where they were asked to tell a story while looking at a wordless picture book. Compared with chronological age- and verbal IQ-matched typically developing children, those with autism or Asperger syndrome did not differ in frequency or range of evaluative devices such as intensifiers and attention-getters. Similarly, it appears that narrative skills are relatively unimpaired also in children with autism who have lower verbal and cognitive abilities (Capps et al., 2000). Capps et al. (2000) examined narrative skills of children with autism, children with developmental delays, and typically developing children using storybook narratives (the groups were matched for language ability). They found several group differences between autistic and typically developing groups; for example, children with autism and children with developmental delays used a more restricted range of evaluative devices such as references to characters’ internal states, but not between autistic and developmental delays groups. The fact that narrative skills are relatively unimpaired in these children with ASD might be due to the demand of the storybook narrative task. In this task, individuals did not need to respond to what the listeners said but were asked to speak what they wanted. Therefore, they might not need to recruit impaired skills such as understanding the intention of others.

While previous studies focusing on storybook narratives revealed relatively unimpaired performance by children with ASD, it appears individuals with ASD tend to show more deficits in interactive conversation, where more complex social communicative skills are needed, such as taking into consideration another’s state of mind and making conversation relevant to the topic. Paul et al. (2009) observed conversational behaviors with examiners, and found that, compared with chronological age-matched typically developing counterparts, adolescents with Asperger syndrome had more difficulty commenting pertinent to the topic, taking into account the listener’s knowledge background, providing the proper amount of information, and maintaining a reciprocal conversational exchange. Also, Capps et al. (1998) observed informal conversation about vacationing, friends, and school, and found that children with autism more often provided no response to comments by the other person, less often gave new and relevant information on an ongoing topic, and more often made bizarre or idiosyncratic responses, compared with language age-matched children with developmental delays.

Similar to ASD, for individuals with WS, vocabulary ability seems to be the strongest area among their language abilities (Brock, 2007). Bellugi et al. (2000) revealed that vocabulary age with WS was significantly better relative to what would be expected from their overall mental age. Unlike an earlier claim that the grammar ability of individuals with WS was intact (Pinker, 1999), the level of grammar ability is below their chronological age, and is thought to be almost on par with their overall mental age (Karmiloff-Smith et al., 1997; Phillips et al., 2004; Brock, 2007). Compared to other aspects of language, studies regarding pragmatic language of individuals with WS are quite few. The debate concerning whether and how much pragmatic language ability with WS is impaired continues. Recently, however, evidence has been accumulating suggesting it is atypical and impaired.

As with ASD, individuals with WS appear to show relative strength in providing narratives, but have deficits in conversation that requires taking others into account. Reilly et al. (2004) analyzed how children with WS told a story while looking at a picture book. They found that, as compared to children with specific language impairment (a developmental disorder diagnosed on the basis of difficulties with language in a child who is otherwise developing normally in the absence of any obvious cause) and typically developing children, children with WS produced a greater proportion of social engagement devices, such as sound effects and audience hookers. Also, Lacroix et al. (2007) found that children and adolescents with WS showed relatively good performance during narrative production, but produced fewer utterances, played a weaker role, and less often satisfied the partner’s requests during conversation, compared to mental age-matched typically developing children.

Recent studies have shown that individuals with WS have difficulty in other areas of pragmatic language. Laws and Bishop (2004) used the Children’s Communication Checklist (Bishop, 1998) or a modified version of it for parents or teachers of individuals with WS, Down syndrome, or specific language impairment. They revealed that the WS group scored well below the cut-off score at which individuals are categorized as having pragmatic difficulties, while the Down syndrome group and the specific language impairment group both scored at about the cut-off. In addition, the WS group showed difficulties especially with the inappropriate initiation of conversation and the use of stereotyped conversation, and scored lower on these subscales than both the Down syndrome group and the specific language impairment group. Moreover, individuals with WS also have difficulty in making comments relevant to the topic of conversation, sharing their knowledge background with the other person, and repairing communication breakdowns. Stojanovik (2006) investigated the social interaction abilities of children with WS using semi-structured conversations. She found that, compared to children with specific language impairment, children with WS were more likely to produce longer verbal responses, but they were less likely to include new information in their replies. Asada et al. (2010a) found that, while children with WS, in general, showed the same amount of language as verbal mental age-matched typically developing children, children with WS showed less attention-sharing communication. Another study reported that children with WS had difficulty in clarifying what they meant when the listener did not understand them, compared to mental age-matched typically developing children (Asada et al., 2010b). Also, John et al. (2009) reported that the skill of repairing communication breakdowns was related to performance on false belief tasks, suggesting that the pragmatic language deficits with WS relates to their social cognitive ability level.

To the best of our knowledge, there is only one study that directly compares the pragmatic language ability of people with ASD to those with WS. Philofsky et al. (2007) used the Children’s Communication Checklist Second Edition (Bishop, 2003) for parents, and directly compared the results for children with ASD with that of children with WS. They found that overall pragmatic functioning, that is, the sum of the pragmatic language subscales of inappropriate initiation, stereotyped language, use of context, and non-verbal communication, was more severely impaired with ASD than with WS. However, the extent of difficulties in the inappropriate initiation of conversation and the use of context did not significantly differ between the groups, although children with WS were rated as more impaired than children with ASD on the inappropriate initiation subscale.

Taken together, the overall language profile of relative strengths (e.g., vocabulary) and weaknesses (e.g., pragmatics) is alike between ASD and WS individuals. Within pragmatic language ability, both individuals with ASD and WS have relatively unimpaired narrative skills, but difficulty with communication involving more social communicative demands, such as taking into account another’s mental state and commenting relevant to the topic at hand, although individuals with ASD generally are more impaired than individuals with WS.

The fact that ASD and WS individuals showed similar profiles on communicative skills might be due to social cognitive deficits. Some theorists on pragmatics have claimed that social cognitive skills are needed for pragmatic language comprehension (Sperber and Wilson, 1987), and indeed for both individuals with ASD and WS a direct relationship between pragmatic language ability and social cognitive skills was found in several studies (Happé, 1993; Surian et al., 1996; Hale and Tager-Flusberg, 2005; John et al., 2009). Studies of language acquisition in typical development suggest that vocabulary ability is achieved in several ways, such as inferring speaker intention and use of the mutual exclusivity rule (i.e., every object has only a single label; Markman and Wachtel, 1988; Baldwin, 1995). One study revealed that children with autism learned new words through mutual exclusivity but not by inferring speaker intention (Preissler and Carey, 2005). Therefore, in ASD, vocabulary might be acquired by using only restricted strategies compared with those of typically developing children. We still do not know whether this leads not only to late onset of language but also to later pragmatic language deficits. People might have to learn vocabulary linking to social context where it is used, or learn it while inferring speaker intention in order to use it appropriately. Future studies should explore whether language acquisition without using social cognitive skills would lead to pragmatic language deficits in ASD and WS.

## Conclusion

Although further cross-syndrome direct comparison studies between ASD and WS are needed, our conclusion regarding the similarities and differences between these two disorders are as follows. (1) Individuals with these disorders show quite contrasting phenotypes with respect to face processing (i.e., preference to faces and eyes) and sociability (i.e., interest in and motivation to interact with others). (2) Although the ADOS and a direct comparison study of pragmatic language ability suggest more deficits with ASD, individuals with WS are similarly impaired with regard to social cognition and communicative skills. Focusing only on sociability, ASD and WS can be viewed as polar opposite disorders (e.g., Jones et al., 2000). However, as we have noted, recent studies have discovered a number of similarities between them, and a growing body of evidence suggests a need to focus on the shared characteristics of these disorders.

There are some limitations of this review. The first has to do with diagnostic categories, levels of functioning, and severity of symptoms. While focusing on a comparison of ASD and WS, we were not able to take a close look at differences according to subcategories on ASD, or to thoroughly consider related levels of functioning (e.g., IQ) or the severity of symptoms. Second is the lack of availability of good comparison group studies focusing on either ASD or WS. That is, some studies recruited only typically developing control group and others used more than one control group, such as individuals with intellectual disabilities without ASD or WS in addition to typically developing controls. Third is the developmental effect of age. According to a report on the anatomical development of the amygdala, compared to typically developing individuals, the volume of the amygdala in individuals with ASD is larger in childhood but not so in adolescence (Schumann et al., 2004). Therefore, symptom expression that relates to amygdala functioning could vary across age ranges due in part to maturation. Recently, research methodology focusing on age-related changes has been utilized for developmental disorders (e.g., Annaz et al., 2009; Thomas et al., 2009). More studies using such a developmental trajectory analysis are needed.

Knowing more about the similarities and differences between these two well-documented disorders could contribute much to both academic and clinical endeavors. First, developmental theory could be more refined. As South et al. (2011) hypothesized, early inadequate motivation for social engagement would lead to less social experience, which subsequently would contribute to eventual later social-perceptual and social-cognitive deficits. For ASD, this seems to be a reasonable hypothesis. On the other hand, individuals with WS display adequate (and even extraordinary) interest in social engagement, and probably have a lot of social experiences. However, as this integrative review indicates, this characteristic of WS does not necessarily lead to functional social skills. One of the clues to this puzzle may come from a computational model proposed by Triesch et al. (2006). According to them, both excessive (as with WS) and scarce (as with ASD) interest in faces should lead to deficits in gaze-following skills, which are an important component of joint attention ability. This is probably because it is difficult to link the partner’s eyes (or attention) to the target that the partner is attending to in both cases. That means different characteristics of face processing and sociability between ASD and WS could predict the same outcome: deficits in social cognition. The developmental pathway leading to some difficulty could be different between ASD and WS, even if the difficulty looks similar.

However, other components, which were not examined here, might be related to the social difficulty in ASD and WS. Karmiloff-Smith 2008) proposed that low-level attention deficits could lead to higher-level linguistic and cognitive deficits since saccadic eye movements involve following the partner’s focus of attention, which is related to social understanding and language acquisition. There has been evidence on deficits with saccadic eye movement and attentional control both in ASD and WS (Goldberg et al., 2002; Brown et al., 2003; Landry and Bryson, 2004; Van der Geest et al., 2004). Further studies examining the effects on social development both from inside and outside of the social domains will help us to understand how development proceeds in these developmental disorders.

Second, neural substrates of social behavioral phenotypes can be identified. Although brain imaging studies of WS still are few, there is considerable evidence available regarding the neural substrates of social behavioral phenotypes of ASD (see Amaral et al., 2008 for a review). The amygdala is one of the well-studied parts of the brain for both disorders. With ASD, amygdala hypoactivation was found during the task of interpreting emotional states from viewing eyes (Baron-Cohen et al., 1999) and of processing fearful faces (Ashwin et al., 2007). With WS, the volume of the right amygdala is correlated to how “approachable” participants rate faces in an approachability task (Martens et al., 2009). In addition, Meyer-Lindenberg et al. (2005) revealed that, in contrast to typically developing individuals, those with WS showed heightened activation of the amygdala to non-social stimuli, and less activation to social stimuli. Therefore, atypical amygdala functioning might be related to social cognitive impairment and aberrant sociability for both disorders. Given that ASD and WS have similar social cognitive deficits but different aspects of sociability, the amygdala might be differentially impaired or other neural domains linked to the amygdala might be related to these two disorders. However, we should keep in mind that aspects of amygdala functioning still are under debate (e.g., arousal rather than social cognition; South et al., 2011). For ASD, the exploration of neural substrates for impaired social behavioral phenotypes is relatively advanced (e.g., superior temporal sulcus for gaze processing, Pelphrey et al., 2005; medial prefrontal cortex, superior temporal sulcus, and temporal poles for attributing mental states, Castelli et al., 2002). Future studies should examine brain functioning in these areas regarding WS.

Third, intervention techniques could be shared. The impaired social phenotypes that this article revealed both in ASD and WS are with respect to social cognitive and communicative skills. Indeed, many of the intervention techniques for ASD focus on these same areas (e.g., social skills training; see Howlin and Charman, 2011 for a review). As Klein-Tasman et al. (2007) pointed out, intervention techniques for ASD were developed to improve behavioral phenotypes, rather than to address the biological causes of disorders, and therefore these techniques should be helpful for other developmental disorders with similar difficulties. Considering that few interventions have been developed for WS, it might be helpful for clinicians and parents to consider these techniques for WS. However, rigorous trials to test the effectiveness of these techniques for ASD still are few in number (Howlin and Charman, 2011). The determination of the effectiveness of each technique for each disorder is necessary and very important.

Finally, this past decade has seen dramatic improvements in the study of developmental disorders. Hereafter, cross-syndrome studies focusing on social phenotypes other than face processing and brain imaging studies will be beneficial in determining the characteristics of these developmental disorders. We believe such attempts will make further contributions to understanding each disorder, and what intervention techniques might be effective for clinical treatments.

## Conflict of Interest Statement

The authors declare that the research was conducted in the absence of any commercial or financial relationships that could be construed as a potential conflict of interest.
